# Novel perspectives on extracellular vesicles in autoimmune diseases: immunogenicity, inflammation, and immune surveillance

**DOI:** 10.1172/JCI194715

**Published:** 2026-02-02

**Authors:** Yin Zhao, Xing Lyu, Xiuhua Wu, Yu Liu, Na Zhang, Wei Wei, Ming-Lin Liu

**Affiliations:** 1 Department of Rheumatology and Immunology, Tianjin Medical University General Hospital, Tianjin, China.; 2 Department of Nephrology and Rheumatology, The Affiliated Hospital of Yunnan University, Kunming, Yunnan, China.; 3 Department of Family and Community Medicine, Northwestern University Feinberg School of Medicine, Chicago, Illinois, USA.; 4 Department of Family Medicine, University of Massachusetts Chan Medical School-Baystate, Springfield, Massachusetts, USA.; 5 Department of Dermatology, Perelman School of Medicine, University of Pennsylvania, Philadelphia, Pennsylvania, USA.; 6 Corporal Michael J. Crescenz VAMC, Philadelphia, Pennsylvania, USA.

## Abstract

Cells release extracellular vesicles (EVs) with cargo that originates from distinct subcellular compartments, including the nucleus, cytoplasm, and plasma membrane. Given their diverse cargo, EVs play multiple roles in physiology and pathology, including in immune dysregulation and autoimmune pathogenesis. For example, EVs can act as autoantigens by transporting immunogenic molecules from the nucleus or cytoplasm, whereas EVs carrying membrane-bound MHCs from antigen-presenting cells can activate adaptive immunity by presenting self-antigens to T cells. EV-associated cytoplasmic peptidases or proteasomes contribute to immune regulation by modulating antigen processing and presentation. Moreover, EVs also drive inflammatory responses by shuttling a variety of proinflammatory molecules that sustain autoimmune responses. Intriguingly, emerging evidence indicates that EVs might contribute to autoimmune surveillance by activating cytosolic surveillance sensors, modulating immune checkpoints, regulating NK/T cell cytotoxicity, and altering macrophage and DC phagocytosis, representing an exciting and underexplored frontier in autoimmune research. By tackling critical knowledge gaps, this Review explores the emerging roles of EVs and their diverse cargo in driving autoimmune diseases, suggesting new perspectives on their potential as innovative therapeutic targets.

## Introduction

Extracellular vesicles (EVs) are ubiquitous in biological systems, carrying cellular compartment–specific bioactive cargo that determines their physiological and pathological functions, from maintaining health to driving disease (1, 2). Eukaryotic cells maintain compartmentalized homeostasis for fundamental cellular processes, such as genetic regulation, protein synthesis and modification, energy production, and redox control (3–5). During programmed cell death (PCD), the disruption of cellular compartments leads to oxidative stress, posttranslational protein modifications, and cryptic epitope exposure. This process releases EVs loaded with diverse cargo from various subcellular compartments, equipping them to exert diverse biological or pathological effects (6, 7).

EV-associated cargo includes proteins, lipids, nucleic acids, and metabolites (8). Their specific profile is influenced by cell type, cellular state (1, 7), and environmental factors, such as pathogens and toxins. Emerging evidence indicates that EVs are versatile carriers for intercellular communications, enabling autocrine, paracrine, and endocrine signaling by transporting cargo that modulates target cell functions (5, 9, 10). EVs interact with recipient cells through receptor-mediated signaling (11) and cargo transfer, thereby affecting various cellular activities (12, 13).

Given their ability to carry immunogenic and proinflammatory cargo, EVs are increasingly recognized for their involvement in dysregulated immune responses and autoimmune disorders (1, 2, 7, 9, 14–16). Intriguingly, immune surveillance, a concept originally described in cancer biology (17), may also be involved in autoimmune diseases (18–20). However, the mechanistic contribution of EVs to immune surveillance dysregulation in autoimmune pathogenesis remains undocumented. This Review synthesizes current evidence on the immunogenic, proinflammatory, and immune surveillance properties of EVs and their implications in autoimmune diseases.

## EV generation and PCD

EVs are highly heterogeneous in size, shape, origin, cargo, and biological function (21). Based on size and biogenesis, they are classified into the following categories: microvesicles (MVs; 100–10,00 nm), formed through plasma membrane budding (15); exosomes (30–100 nm), generated within endosomal multivesicular bodies (MVBs) and released via exocytosis (22); and apoptotic bodies (1,000–5,000 nm) (1, 23), released during late apoptosis, containing organelles and fragments of the disintegrated nucleus (1, 15, 24). Notably, membrane vesiculation and blebbing during apoptosis lead to the release of various types of apoptotic cell–derived EVs, including exosome-like vesicles, MVs, apoptotic bodies, fragmented beaded apoptopodia, and the remnant apoptotic cell body (2, 9, 14, 23).

EVs can also be released during other forms of PCD (2, 25). Pyroptotic cells release EVs that carry gasdermin D (GSDMD), caspase-1, Fas-associated death domain (FADD), and cytokines, which results in promotion of inflammatory responses (2, 26). Necroptotic cells release EVs (0.2–0.8 μm) that carry phosphorylated mixed lineage kinase domain-like (MLKL) and endosomal sorting complex required for transport III (ESCRT III) components (27, 28), with exposed phosphatidylserine (PS) that promotes immune recognition (27). Unlike apoptotic bodies, necroptotic EVs contain minimal DNA/RNA (28). NETosis, a unique proinflammatory neutrophil death mechanism characterized by neutrophil extracellular trap (NET) formation (29, 30), also releases EVs (31). Ferroptosis, an iron-dependent form of cell death driven by lipid peroxidation (32), releases ferroptosis-dependent EVs that can transport iron-loaded ferritin to recipient cells, promoting DNA damage and carcinogenesis (25).

As a fundamental biological process, cell death can be immunologic or nonimmunologic, depending on the specific cellular mechanisms, the cellular components released, and the surrounding microenvironment (33, 34). Under pathological conditions, multiple types of PCD — including apoptosis, pyroptosis, necroptosis, NETosis, and ferroptosis — serve as potent sources of damage-associated molecular patterns (DAMPs) (1, 2, 14). These PCD pathways often converge into an immunogenic cell death phenotype, which is characterized by the release of DAMPs and the exposure of cryptic nuclear and cytoplasmic autoantigens (33, 34) following the nuclear and plasma membrane breakdown and subcellular compartment disruption. Furthermore, the dynamic membrane budding and remodeling during PCD drives EV formation (35, 36), enabling the selective packaging of immunogenic molecules into EVs. Consequently, PCD-derived EVs can transport various immunogenic cargo, thereby contributing to autoimmune pathogenesis (37) and immune surveillance (38, 39).

## Mechanisms of cargo sorting into EVs

During EV biogenesis, molecular components from the nucleus, cytoplasm, and plasma membrane can be selectively incorporated into EVs. The nuclear envelope, composed of lipid bilayers and a meshwork of lamin protein, may rupture under certain conditions, leading to the release of immunogenic and proinflammatory nuclear contents (30, 31, 40). Nuclear envelope budding exports nuclear molecules (41, 42) to the cytoplasm and plasma membrane, enabling their release via EVs (14, 43, 44). In activated macrophages, high-mobility-group box 1 (HMGB1) can exit the nucleus through chromosome region maintenance-1–mediated (CRM1-mediated) export, incorporate into the plasma membrane, and then be released via EVs (14). Damaged nuclear DNA can form cytoplasmic micronuclei (45), which may be packaged into EVs (43, 44) and trigger type-1 IFN (IFN-I) production in innate immune cells (31, 40). Moreover, EVs can encapsulate cytoplasmic molecules and organelles, including mitochondria, the Golgi apparatus, endoplasmic reticulum, and lysosomes (46, 47). During EV biogenesis, the cytoplasm acts as a reservoir for nuclear autoantigens (48), DAMPs (14), and micronuclei (45), which are subsequently packaged and released within EVs. Furthermore, plasma membrane proteins (receptors, integrins, ion channels, and enzymes) (14, 49) can be released through membrane vesiculation during MV formation (2, 14). Additionally, extracellular molecules like complement proteins (50) and lipoproteins (51) can be incorporated into EVs, forming a protein corona (51).

The mechanisms governing the export of nuclear/cytoplasmic contents, incorporation of plasma membrane/extracellular molecules, and selective cargo packaging into EVs remain largely unclear. However, recent studies indicate that EVs package subcellular cargo selectively, rather than randomly, through several interconnected mechanisms (21, 52). (a) The ESCRT machinery (ESCRT-0/-I/-II/-III) is essential for sorting cargo into MVBs, which are then released as EVs (52). (b) Lipid rafts are cholesterol/sphingolipid-rich microdomains that contain glycosylphos-phatidylinositol-anchored proteins, signaling molecules, and MHC (53). Lipid raft–associated ceramides induce membrane curvature and budding, while lysophospholipids facilitate selective incorporation of raft-associated molecules into EVs during vesiculation (54). (c) Tetraspanins, a conserved group of transmembrane proteins, form tetraspanin-enriched microdomains by interacting with proteins, glycoproteins, and lipids (55), facilitating EV cargo sorting by clustering molecules and recruiting EV biogenesis machinery (56). Depletion of specific tetraspanins impairs EV release (54). (d) RNA-binding proteins (RBPs) like the heterogeneous nuclear ribonucleoprotein (hnRNP) family, Y-box binding protein 1 (YBX1), and annexin A2 (ANXA2) are crucial for the selective packaging of both coding and noncoding RNAs into EVs (52). Multiple RBPs can synergistically facilitate RNA sorting (57). (e) Posttranslational modifications (PTMs), like ubiquitylation, glycosylation, and phosphorylation, can influence selective protein sorting into EVs (52, 58), though their roles in EV biogenesis require further investigation (58). The sorting mechanisms for mitochondrial DNA (mtDNA) (52) and nuclear molecules into EVs or apoptotic bodies, respectively, remain incompletely elucidated. In late-stage apoptosis, diminishing cellular regulation may result in the incorporation of nuclear molecules into apoptotic bodies via both active and passive mechanisms, though their relative contributions remain unclear.

## EVs and their cargo molecules in autoimmune pathogenesis

**Figure 1:**
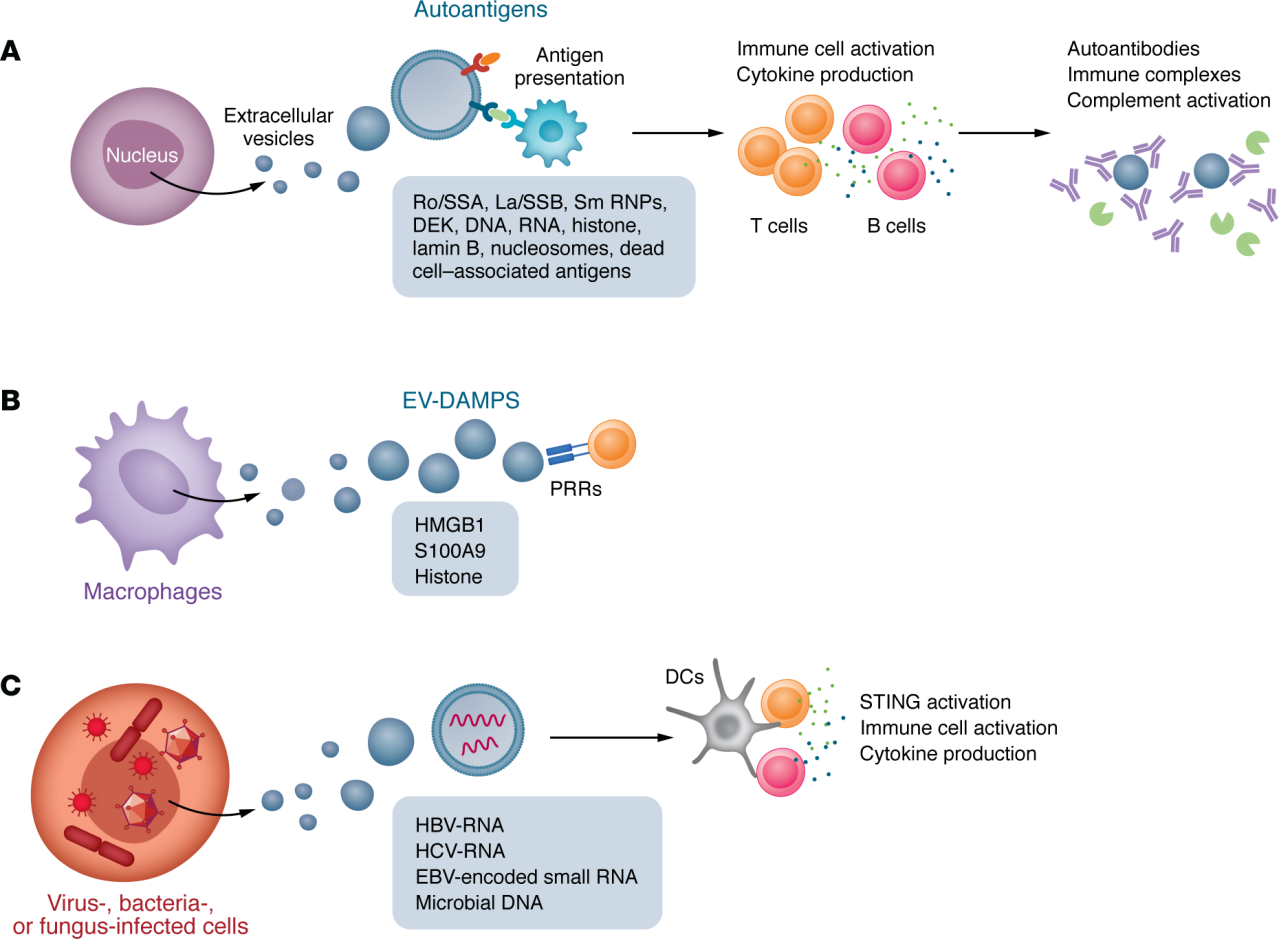
EVs with nuclear cargo molecules in autoimmune diseases. (**A**) EVs package nuclear-derived molecules, including Ro/SSA, La/SSB, Sm RNPs, DEK, DNA, RNA, and lamin B, which serve as immunogenic autoantigens. These molecules promote B cell activation, differentiation, and autoantibody production, while also activating T cells through antigen presentation. EV-associated nuclear autoantigens can further induce immune complex formation, contributing to autoimmune pathogenesis. (**B**) EVs can also transport nuclear DAMPs, such as HMGB1, S100A9, and histones, which activate immune cells via pattern recognition receptors (PRRs). (**C**) Notably, infected cells can release EVs containing microbial nucleic acids that stimulate immune cells to produce proinflammatory cytokines through various signaling pathways, including STING. SSA, Sjögren’s syndrome A; SSB, Sjögren’s syndrome B; RNP, ribonucleoprotein; DAMPs, damage-associated molecular patterns; HMGB1, high mobility group box 1; STING, stimulator of interferon genes.

**Figure 2:**
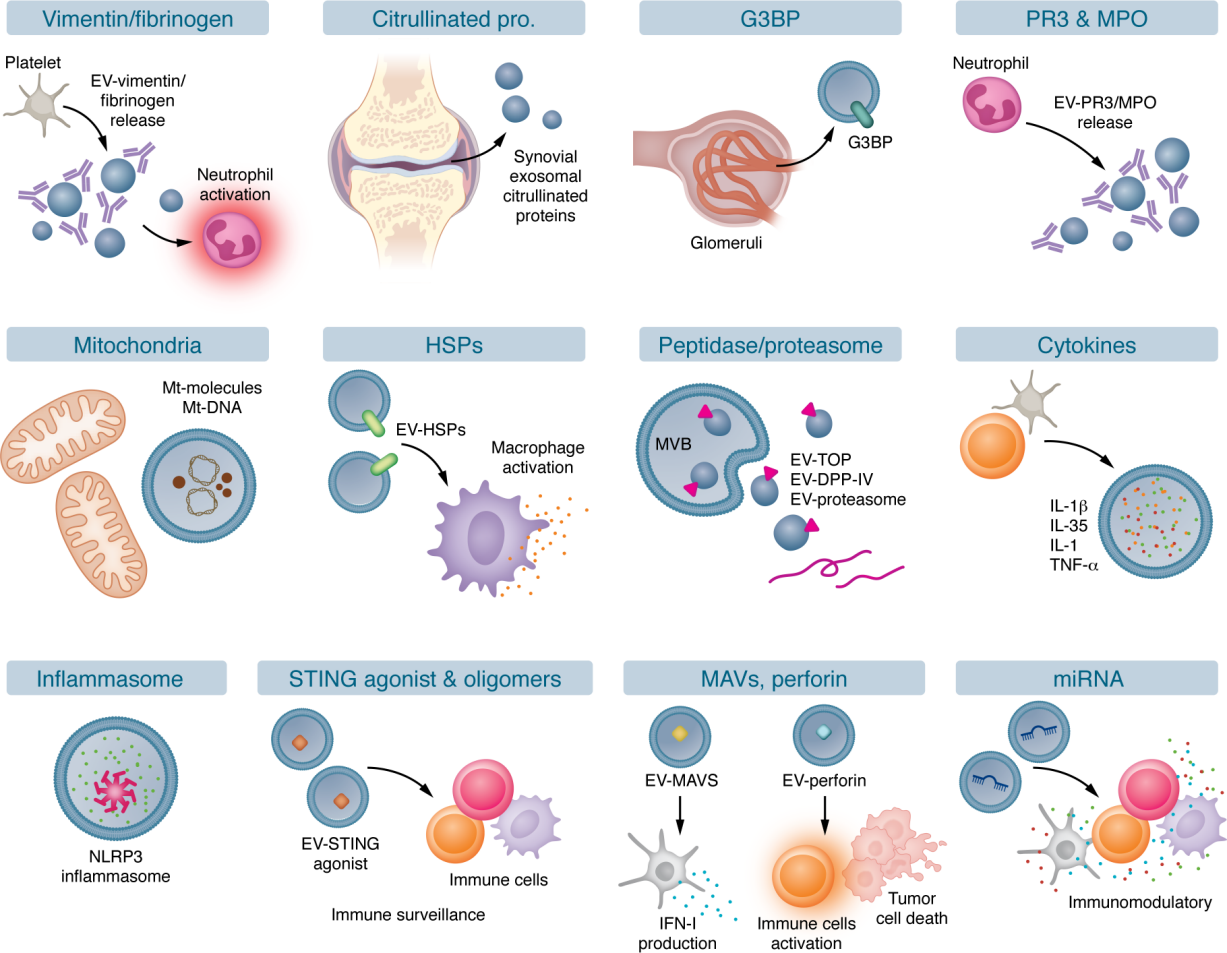
EVs with cytoplasmic cargo molecules in autoimmune diseases. EV-associated cytosolic autoantigens, including vimentin, fibrinogen, citrullinated proteins, G3BP, PR3, and MPO, can promote B lymphocyte activation, autoantibody production, and immune complex formation through binding with autoantibodies, thereby contributing to autoimmune pathogenesis. EVs harboring cytosolic DAMPs, such as mtDNA and HSPs, can stimulate inflammatory cytokine release from innate immune cells and trigger intrinsic immune surveillance. Furthermore, EV-associated cytosolic molecules, including proteasomes, TOP, and DPP-IV, can modulate antigen processing and presentation. EV-associated inflammatory cytokines and inflammasome can activate inflammatory signaling pathways. EVs containing STING oligomers/agonists and perforin are involved in autoimmune surveillance. EV-associated MAVS can promote DC production of IFN-I. In addition, EVs can transport miRNAs involved in immunomodulation. G3BP, galectin-3–binding protein; PR3, proteinase 3; MPO, myeloperoxidase; DAMPs, damage-associated molecular patterns; mtDNA, mitochondrial DNA; TOP, thimet oligopeptidase; DPP-IV, dipeptidyl peptidase IV; STING, stimulator of interferon genes; MAVS, mitochondrial antiviral signaling protein; Mt-molecules, mitochondrial molecules.

**Figure 3:**
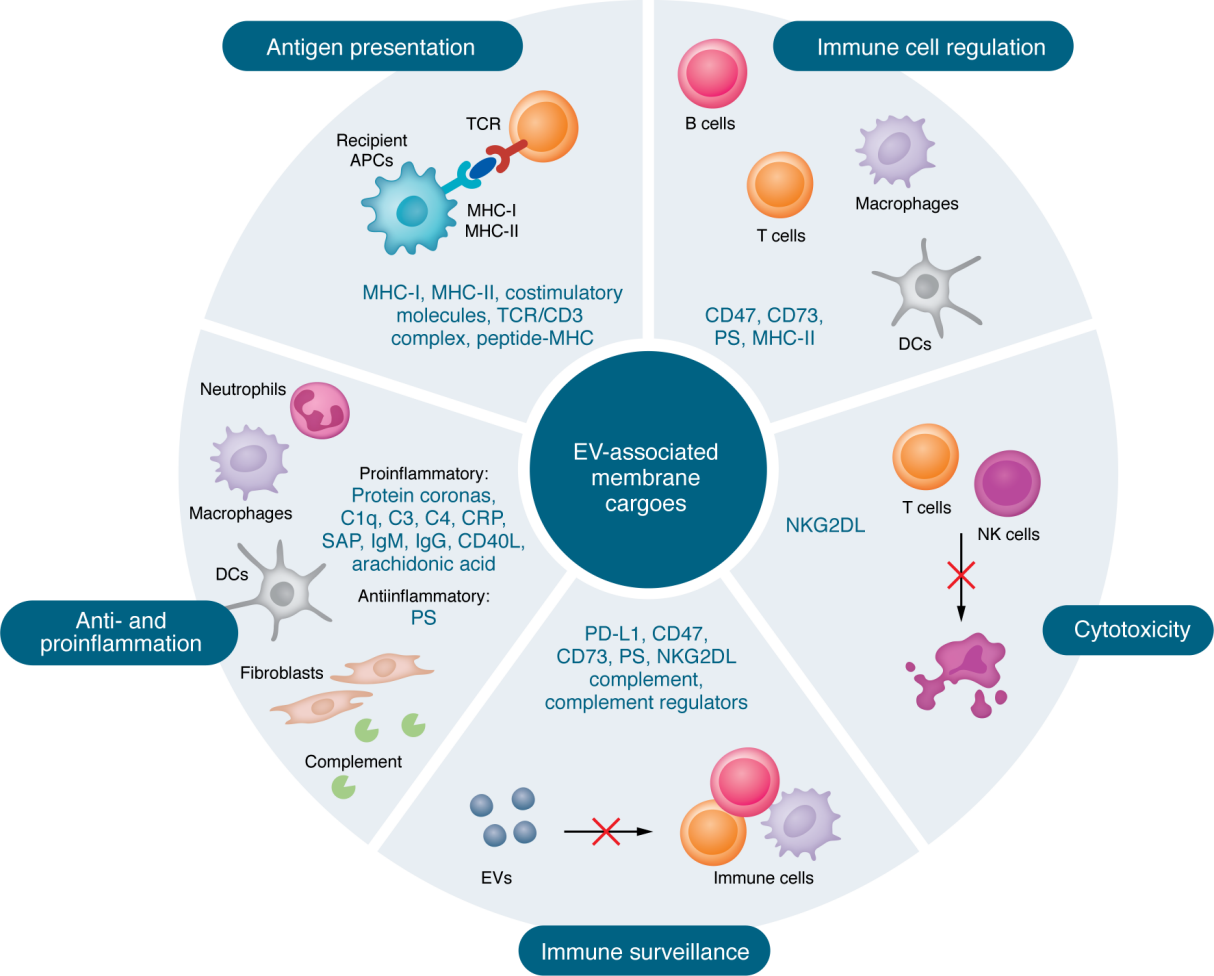
EVs with cell membrane and extracellular cargo molecules in autoimmune diseases. EVs can participate in antigen presentation by harboring MHC-I, MHC-II, costimulatory molecules, TCR/CD3 complex, and peptide-MHC complexes, which can activate immune responses and potentially contribute to autoimmune diseases. By carrying diverse membrane molecules, EVs can regulate the activation, proliferation, differentiation, and other functions of various immune cells, including macrophages, DCs, CD8^+^ T cells, Tregs, and B cells. EVs containing NKG2D ligands can inhibit the cytotoxic activity of NK and T cells. EVs containing PD-L1, CD47, CD73, PS, NKG2D ligands, complement, and complement regulators are involved in the process of immune surveillance. Finally, EV-associated protein coronas, complement components, activators/regulators, immunoglobulins, CD40L, and membrane lipids as well as PS may exert pro- or antiinflammatory effects depending on their cargo molecules. TCR, T cell receptor; NKG2D, NK group 2D; PD-L1, programmed cell death ligand 1; PS, phosphatidylserine.

Growing evidence demonstrates that EVs contribute to autoimmune pathogenesis by transporting immunogenic cargo from nuclear, cytoplasmic, and plasma membrane components (Figures 1, 2, and 3). Dying cells release EVs enriched with danger signals, immunogenic molecules, and immunostimulatory factors (33, 34), which can disrupt immune tolerance, trigger aberrant immune responses, and accelerate autoimmune pathogenesis. EVs may promote autoimmunity through multiple mechanisms, including transport of immunogenic autoantigens (59–61), antigen processing (39) and presentation (62, 63), and modulation of Tregs/Th17 balance (64, 65). Here, we review the current understanding of how EV-associated cargo from distinct subcellular origins contributes to autoimmune pathogenesis via interconnected mechanisms.

## EV-associated nuclear molecules

Nuclear molecules are normally hidden from the immune system, so when they are released with EVs they may be misinterpreted as foreign and trigger autoimmune responses. EVs can transport nuclear autoantigens to lymphatic organs, where antigen-presenting cells (APCs) process and present them, activating adaptive immunity (66). EV-associated nuclear DNA may function as a self-antigen to activate autoreactive B cells (59) and stimulate plasmacytoid DCs (pDCs) to produce IFN-I (67). Furthermore, both DNA and IFN-I promote extrafollicular B cell activation (68) and autoantibody production, establishing a self-perpetuating cycle that sustains autoimmunity (68). Supporting this model, in Dnase1l3-deficient mice, impaired DNA degradation in apoptotic MVs triggers anti-DNA antibody production, accelerating lupus pathogenesis (60). Clinically, patients with DNASE1L3 deficiency develop monogenic systemic lupus erythematosus (SLE) characterized by elevated levels of MV-associated DNA (69). Notably, over 50% of patients with sporadic SLE and lupus nephritis exhibit reduced DNASE1L3 activity attributable to neutralizing autoantibodies, further implicating impaired MV-DNA clearance in autoimmune pathogenesis (69). Furthermore, patients with SLE exhibit elevated levels of IgG-, IgM-, and C1q-bearing EVs. Importantly, IgG^+^ EVs correlate with anti-dsDNA antibodies and complement activation (70), two well-established biomarkers of active lupus.

Other nuclear autoantigens, including ribonucleoproteins (Ro/SSA, La/SSB, and Sm/RNPs) (61, 71), RNA (71), HMGB1 (14), histone H3, lamin B1 (72), and DEK (73), can be incorporated into EVs or apoptotic bodies (Figure 1 and Table 1), with significant clinical implications. In patients with SLE, circulating HMGB1-containing nucleosomes contribute to autoimmune pathogenesis (74). These nucleosomes can induce DC maturation in vitro and trigger autoantibody production in vivo when administered to mice (74). In patients with arthritis, exosome-associated DEK, DEK autoantibodies, and immune complexes (ICs) have been detected in synovial fluid, with DEK acetylation critically contributing to its antigenicity (73). DEK-containing exosomes may also contribute to joint inflammation by recruiting neutrophils, NK cells, and CD8^+^ T lymphocytes, thereby amplifying inflammatory and immune responses (48).

The presence of EV-associated nuclear autoantigens may be crucial for the progression of autoimmune diseases, suggesting potential new targets for clinical intervention. This process is further complicated by PTMs, such as citrullination, carbamylation, methylation, acetylation, and peroxidation, which contribute to the formation of neoantigens in EVs that can break immune tolerance (7, 73, 75). However, while these nuclear autoantigens, including DNA, citrullinated histones (75) and lamin B (30, 31) are clearly presented in the released NETs, it remains unclear whether EVs released from NETotic neutrophils also carry them.

## EV-associated cytoplasmic molecules serve as autoantigens

Beyond nuclear autoantigens, EVs can also transport cytoplasmic components with autoantigenic properties (76–79) (Table 2). In patients with rheumatoid arthritis (RA), platelet MVs in synovial fluid can transport autoantigenic citrullinated vimentin and fibrinogen, which can form ICs with anti-citrullinated peptide antibodies, potentially amplifying synovial inflammation via neutrophil activation and leukotriene production (76). Similarly, another study reported that synovial exosomes from patients with RA carry citrullinated fibrin-derived peptide autoantigens (79). These findings position EVs as pivotal players in RA pathogenesis by delivering immunogenic citrullinated autoantigens (76, 79). Patients with SLE demonstrate elevated levels of circulating galectin-3–binding protein^+^ (G3BP^+^) MVs, which colocalize with ICs in renal tissues (78), suggesting their direct contribution to lupus nephritis pathogenesis through promoting renal IC deposition. Patients with antineutrophil cytoplasmic antibody–associated vasculitis show elevated levels of circulating proteinase 3 (PR3) autoantigen (80), which also present in neutrophil-derived MVs (77), implicating EVs in autoantigen dissemination and disease pathogenesis. Together, these clinical and experimental findings underscore the importance of EV-associated cytoplasmic autoantigens in driving autoimmunity and disease progression by breaking immune tolerance and promoting pathogenic autoantibody production (Figure 2).

### EV-associated proteases and proteasomes in antigen presentation.

Emerging evidence demonstrates that EVs can modulate antigen presentation. Platelet EVs from human blood have been found to carry proteasomes that can process proteins for antigen presentation to CD8^+^ T cells in mice (39), highlighting their contribution to adaptive immunity. Furthermore, apoptotic exosomes containing 20S proteasome complexes have been shown to regulate autoantibody production through proteasome-mediated degradation, contributing to allograft rejection in mice (81). Cytoplasmic thimet oligopeptidase (TOP) regulates autoimmunity by degrading intracellular antigenic peptides (82), thereby modulating MHC-I antigen presentation and T cell activation (83). Interestingly, TOP retains its enzymatic activity when released via EVs (84, 85), implying its potential role in EV-mediated antigen processing and immune modulation. Moreover, dipeptidyl peptidase IV (DPP-IV), known to promote T cell activation via CD86 upregulation on APCs (86), has been identified in human EVs (87, 88), suggesting its potential involvement in autoimmune pathogenesis (89). Further studies are required to fully elucidate the mechanistic roles of EV-associated proteasomes, TOP, and DPP-IV, and their therapeutic potential (Figure 2 and Table 2).

## EVs with plasma membrane molecules in autoimmunity

Beyond antigen processing, exosomes/MVs from APCs can activate T cells in experimental mice by directly presenting antigens to them via MHC-I, MHC-II, and the costimulatory molecules CD80 and CD86 (49, 62) (Figure 3). These EVs also mediate intercellular transfer of functional peptide-MHC complexes between DCs, efficiently activating antigen-specific naive CD4^+^ T cells in mice when administered in vivo (49). MHC-II–bearing EVs from antigen-presenting B cells have been shown to activate CD4^+^ T cells, promoting autoimmunity by driving effector T cell differentiation, B cell proliferation, and immunoglobulin class-switching (62). Conversely, exosomes from activated T cells carry TCR/CD3 complex and adhesion molecules, enabling them to facilitate intercellular communication and signal delivery to cells that express peptide-MHC complexes (90) (Table 3).

Whereas classical T cell activation requires direct cell-to-cell contact (5, 91), a bidirectional EV-mediated exchange between APCs and T cells represents a distinct, noncanonical pathway for immune communication. Notably, EV-mediated T cell activation may not fully replicate the sustained, highly coordinated signaling of the classical immunological synapse (5). Nevertheless, this process potently modulates immune responses by amplifying antigen-specific signals and disseminating information to multiple T cells or neighboring APCs. Thus, EVs play a nuanced regulatory role, complementing rather than replacing conventional T cell activation.

## EVs and Tregs in autoimmune diseases

Tregs are essential for immune tolerance and preventing autoimmune diseases (92). Treg-derived exosomes from human plasma or cell cultures carry ecto-5′-nucleotidase enzyme CD73, which generates immunosuppressive adenosine (63, 93). Moreover, Treg-derived EVs may mediate T cell suppression in vivo by transporting immunosuppressive IL-35, which can coat bystander lymphocytes and lead to their exhaustion (94). Treg-EVs can also mediate immune suppression by transferring specific microRNAs, including miR-150-5p and miR-142-3p, which induce tolerogenic DCs by modulating their IL-10 and IL-6 release (95). In patients with multiple sclerosis, Treg-EVs demonstrate impaired immunosuppressive function compared with those from healthy individuals, a deficit attributed to their reduced miR-142-3p content (96). In mice, Treg-derived exosomes carrying Let-7d miRNA have been shown to suppress Th1 cell proliferation and cytokine secretion in vivo, thereby mitigating systemic inflammation, contributing to suppression and prevention of systemic diseases (97).

Th17 cells oppose Tregs by promoting inflammation in autoimmune disorders. The Treg/Th17 balance maintains immune homeostasis, while its disruption drives autoimmunity (98, 99). Administration of EVs from TGF-β–induced Tregs (100) or gingival mesenchymal stem cells (MSCs) (64) has been shown to improve arthritis in mice by suppressing T cell proliferation and restoring Th17/Treg balance via transferring miR-449a-5p (100) or miR-148a-3p (64), respectively. Similarly, human bone marrow MSC-EVs (65) can improve periodontitis by restoring the Th17/Treg balance in mice via miR-1246 (65). Moreover, labial gland MSC-derived EVs can attenuate experimental Sjögren’s syndrome (SS) in mice by modulating Th17/Treg balance through miRNA let-7f-5p (101), offering a promising target-driven approach for the treatment of SS.

All of the above studies indicate that Treg-EVs and MSC-EVs may mediate immune regulation by transferring key components, including CD73, IL-35, and various immunomodulatory miRNAs, to T cells or DCs, thereby suppressing T cell activity or restoring Th17/Treg balance (Tables 2 and 3).

## EVs and their cargo molecules in autoimmune inflammation

Inflammation plays a critical role in sustaining autoimmune responses and mediating end-organ damage in autoimmune diseases. EVs transport inflammatory cargo, including nuclear and cytoplasmic DAMPs, cytokines, and other mediators, driving autoimmune inflammation (14, 102–111). EVs carrying membrane cargo may either promote or suppress inflammation (112–115). Additionally, EVs can incorporate extracellular molecules to form a protein corona that amplifies their inflammatory potential (50, 51, 116).

## EV-associated nuclear molecules and inflammatory responses

Studies demonstrate that EVs transport various nuclear DAMPs, including nucleic acids (102–104), HMGB1 (14), S100A9 (105), and histones (117), which can activate innate immune responses (Figure 1). EVs from activated macrophages transport nuclear HMGB1 (14), and blood from patients with SLE contains HMGB1-associated nucleosomes (74), which have been shown to stimulate DCs and macrophages, thereby amplifying cytokine production and autoimmune responses in mice (74). Additionally, intraperitoneal injection of histone-containing EVs from *P*. *gingivalis*–infected macrophages can induce lung inflammation and alveolar destruction in mice (117), indicating their role in remote inflammatory responses. These in vivo findings suggest that EV-associated HMGB1 and histones are critical pathogenic drivers of systemic inflammation and immune dysregulation (74, 118).

EVs can carry host-derived nuclear DNA (60), which can trigger pDCs (67) or macrophages (119) to produce IFN-I and other cytokines. Emerging evidence reveals they also transport microbial-derived DNA (120–122), highlighting their importance in host-microbiome interactions in autoimmune pathogenesis. Virus-infected cells release EVs containing viral DNA/RNA, triggering various immune responses. Exosomes from hepatitis B/C virus–infected (HBV/HCV-infected) cells can stimulate NK cells or pDCs to produce IFN-γ (102) or IFN-I (103), respectively. Similarly, exosomes from latent Epstein-Barr virus–infected (EBV-infected) B cells contain small RNAs that trigger DCs to produce IFN-β (104). Additionally, EV-packaged bacterial DNA can be delivered to cells, triggering stimulator of interferon genes–mediated (STING-mediated) IFN-I production in mice (123). This mechanism extends to the gut microbiota, where EV-transported microbial DNA can enter the circulation and drive systemic inflammation in distant organs via the cyclic GMP-AMP synthase (cGAS) and STING pathways (124). Notably, fungal DNA within EVs can activate the STING pathway in macrophages, stimulating IFN-I responses (125). The delivery of immunostimulatory nucleic acids via EVs provides a clinically relevant mechanism that bridges common environmental triggers (e.g., infection, cellular damage) to the breakdown of self-tolerance (Table 1).

Therefore, by transporting nuclear DAMPs alongside host and microbial DNA/RNAs, EVs serve as a central link between PCD, host-pathogen interactions, and the initiation of autoimmunity, driving the inflammatory responses that sustain autoimmune pathogenesis.

## EV-associated cytoplasmic cargo molecules in autoimmune inflammation

Normally confined within cells, cytoplasmic DAMPs, like mtDNA (106), HSPs (111), and inflammasome components (109), can be packaged into EVs (Figure 2). Exosomes carrying mtDNA from human blood can stimulate proinflammatory cytokine production, including IL-1β release from microglia (106) and monocytes (126). Additionally, oxidized mtDNA-carrying exosomes from activated T cells have been shown to trigger IFN-I production in DCs via antigen-specific interactions (127). Furthermore, EVs with mitochondrial antiviral signaling protein (MAVS) can also stimulate DCs to produce IFN-β (110). Under cellular stress, cytoplasmic HSP70 translocates to the plasma membrane (128) and is subsequently released with EVs (111). EV-associated HSP70 is 260-fold more effective than free recombinant HSP70 in inducing macrophage TNF-α production in vitro (111), suggesting that membrane-dependent EV delivery significantly enhances HSP70 immunostimulatory capacity (111). HSP70-containing EVs can suppress tumor progression in melanoma and colon carcinoma mouse models by promoting antitumor cytokine (IL-10, TNF-α, and IFN-γ) production and enhancing NK cell activity (129), indicating their role in immunomodulatory and inflammatory responses. Additionally, microglia-derived exosomes can transport apoptosis-associated speck-like protein containing a CARD (ASC), which can induce inflammasome activation and IL-1β production in vitro and in vivo (109).

Contrary to the traditional view of cytokines as freely diffusible molecules, several studies have reported that some cytokines can be transported by EVs, including IL-1β (107) and TNF-α (108). A systematic analysis further identified up to 33 cytokines associated with EVs that can functionally activate target cells (46). By serving as mobile cytokine reservoirs, EVs enhance cytokine stability and enable targeted delivery to distant tissues. EVs that are engineered and loaded with specific cytokines to precisely modulate immunity could offer significant clinical advantages over freely diffusible cytokines (46, 130) (Table 2).

## EV-associated membrane and extracellular cargo in inflammation

EVs transport diverse membrane molecules that exhibit multiple roles (112–115) (Figure 3). CD40 ligand–bearing (CD40L-bearing) EVs have been shown to promote liver inflammation in vivo by triggering macrophage cytokine production (113), while EVs carrying oxidized membrane lipids can induce endothelial activation and vascular inflammation (131). In RA, leukocyte EVs may amplify synovial inflammation by transferring arachidonic acid to fibroblasts, thus driving proinflammatory prostaglandin E2 production (112). Conversely, PS/annexin A1–bearing neutrophil MVs from patients with RA have demonstrated antiarthritic effects in mice by suppressing macrophage activation and promoting TGF-β production (115). Furthermore, PS-exposing EVs have been reported to promote CD8^+^ T cell proliferation and potentiate antiviral immunity in mice, suggesting their therapeutic potential for augmenting adaptive immunity against viral infections (114).

Beyond their intrinsic cargo, MVs may incorporate extracellular components on their surface, including complement (C1q/C3/C4), C-reactive protein (CRP), serum amyloid P (SAP), and immunoglobulins (IgM/IgG) (50). In patients with RA, synovial fluid EVs show significantly higher levels of C1q/C3/C4 than plasma-derived EVs, indicating localized activation of the classical complement pathway within affected joints (50). Similarly, patients with SLE exhibit elevated circulating levels of complement-bearing MVs compared with healthy controls (116). Emerging research reveals that EVs can dynamically form a functional protein corona by incorporating diverse extracellular molecules from surrounding biological fluids — including complement, apolipoproteins A1/B/C3/E, and fibrinogen (51). These corona proteins can enhance EV immunomodulatory functions by boosting DC cytokine production (51), amplifying EVs’ potential to regulate immune responses (132), and facilitating EV-immune cell interactions (133) (Table 3).

## Emerging roles of EVs in autoimmune surveillance

Originally proposed a century ago, the immune surveillance concept postulated that the immune system constantly identifies and eliminates potentially cancerous cells (17, 134). Although initially doubted owing to a lack of evidence, this concept is now widely accepted, and the elevated cancer incidence in immunodeficient humans and mice (134) underscores the critical role of the immune system in controlling oncogenesis. Moreover, this fundamental mechanism is not limited to cancer but extends to a broad spectrum of pathological conditions — including autoimmune disorders, infection, aging, and tissue injury (18–20, 135–140).

The innate immune system, including macrophages and DCs, plays a key role in immune surveillance by continuously detecting tumor-associated antigens (TAAs) (17, 141, 142). These professional APCs process and present TAA fragments to T cells, initiating adaptive immune responses that lead to cancer cell elimination by cytotoxic T cells (17, 141, 142). Furthermore, NK cells contribute to antitumor immunity by eliminating “missing-self” cancer cells that have downregulated TAAs (141), while macrophages can directly destroy cancer cells via phagocytosis (141). Understanding of this foundational mechanism has driven advances of cancer immunotherapies, including chimeric antigen receptor (CAR) T cell therapy (143).

In autoimmune diseases, defective clearance of cells undergoing PCD results in accumulation of immunogenic debris. APCs take up the debris and present self-antigens to T cells, triggering autoreactive T cells to target altered self-cells, which cause tissue damage and autoimmune pathogenesis (18, 144). This process mirrors tumor surveillance (17, 142), where the failed clearance of mutated cells enables tumorigenesis (17, 134). The fundamental distinction is that inadequate immune surveillance allows cancer growth, whereas excessive immune activity targeting altered-self tissues drives autoimmune pathogenesis (Table 4) (7,18,^140^). Interestingly, patients with autoimmune diseases have an elevated risk of developing certain cancers (145), and cancer immunotherapies may trigger autoimmune conditions (146), which in some cases are paradoxically linked to a better cancer prognosis (147). Furthermore, the effectiveness of CAR T therapy in treating B cell malignancies (19) and SLE (148) has sparked interest in targeting autoreactive B cells to treat other autoimmune diseases. This evidence suggests that autoimmunity and cancer are interconnected, both stemming from dysregulated immune homeostasis (149), and share some common components of immune surveillance mechanisms (Table 4).

The recently proposed autoimmune surveillance of hypersecreting mutants hypothesis (18) suggests that autoreactive T cells can help maintain endocrine homeostasis by eliminating hyperactive endocrine cells, implying that autoimmunity may result from a dysregulation of this surveillance process (140). This concept extends to EVs and their cargo, which have been implicated in multiple aspects of immune regulation and surveillance (2, 7, 141, 142).

## EV-associated nuclear cargo in immune surveillance

Like TAAs, EV-associated nuclear autoantigens can be recognized by APCs (60, 67, 127), which, in turn, release cytokines and activate T cells (66, 127, 144). Notably, damaged nuclear DNA, sequestered in micronuclei (45), can be packaged into EVs (43, 44). Upon release, the EV-associated DNA can be recognized as “foreign” by immune cells through cytosolic surveillance sensors, including the cGAS and STING pathways critical for immune surveillance in both cancer and autoimmune diseases (144, 150, 151). Through in vitro and in vivo studies, Mackenzie and colleagues demonstrated that rupture of the micronuclear envelope leads to rapid cGAS recruitment, alerting the cytosolic surveillance sensor and producing IFN-I (150), a key player in autoimmune diseases like SLE. This research suggests that a breakdown in this specific pathway — which is meant to protect cells from foreign DNA — could be a primary trigger for autoimmune diseases.

When nuclear HMGB1 is released from cancer cells, it can initiate an autocrine signaling loop that upregulates immunosuppressive galectin-9, which inhibits NK and T cell antitumor activity and enables the cancer cells to evade immune surveillance (152). HMGB1, which can also be released via EVs (14), has been shown to impair immune surveillance by inhibiting macrophage phagocytic clearance of apoptotic cells through PS binding (153), leading to accumulation of immunogenic cell debris and PCD-derived EVs. Furthermore, HMGB1 can enhance DC uptake of extracellular DNA, thereby promoting cGAS-STING activation and amplifying IFN-I production (154). Thereby, EV-associated nuclear DNA and HMGB1 may contribute to autoimmune diseases (2, 7, 155) by modulating cytosolic surveillance (150) and interfering with dead cell clearance (153), although further research is needed to understand the precise role of nuclear cargo-carrying EVs in autoimmune surveillance and pathogenesis.

## EV-associated cytoplasmic cargo in immune surveillance

Emerging evidence indicates that EVs may play a regulatory role in immune surveillance by modulating cytotoxic T cell and NK cell activity (156). Human NK cell–derived exosomes from blood carry cytoplasmic perforin and granulysin (157–159), which can kill tumor cells (157, 158) and activate T cells (157, 158) in vitro, indicating their potential involvement in immune surveillance. Moreover, NK cell–derived EVs have been shown to shuttle microRNAs that enhance T cell activation and promote monocyte differentiation into DCs with upregulated MHC-II/CD86 expression, thereby increasing their antigen presentation capacity (160). Notably, delivering NK-EVs enriched with specific miRNAs (miR-10b-5p, miR-92a-3p, and miR-155-5p) to mice can recapitulate these immunomodulatory effects on T cell responses (160). Furthermore, Treg-derived EVs can transport immunomodulatory miRNAs that suppress T cell and DC activation (95, 96, 161), demonstrating their potential role in maintaining immune tolerance and surveillance.

EVs carrying both mtDNA and PD-L1 have been shown to promote tumor progression in mice by shaping an immunosuppressive tumor microenvironment with IFN-I and IL-6 production from macrophages, while simultaneously suppressing T cell immunity (162). Notably, EVs carrying STING oligomers have been reported to suppress innate immune responses, serving as a negative feedback mechanism to prevent STING pathway overactivation (163). However, intratumoral injection of STING agonist–loaded EVs in mice can potently enhance antitumor immune surveillance by stimulating CD8^+^ T cell activation and IFN-I production (164). Therefore, EVs play a complex, context-dependent role in regulating STING signaling.

## EV-associated membrane or extracellular cargo in immune surveillance

Exposed on apoptotic cells and MVs (7, 165), PS serves as an “eat-me” signal for macrophage phagocytosis (166). This process is essential for maintaining immune homeostasis, playing a critical role in promoting antitumor immunity (167, 168) and preserving immune tolerance (169, 170) (Table 4). Disruption of this process — due to excessive apoptosis, dysregulated PS exposure on tumors, or elevated levels of PS^+^ EVs — can overwhelm immunosurveillance, enabling tumor evasion and progression (167, 168) or breaking immune tolerance and triggering autoimmunity (170). In SLE, PS-bearing MVs may competitively inhibit macrophage phagocytosis of apoptotic cells (171), resulting in impaired clearance of dying cells (172) and a loss of immune tolerance. Interestingly, recent research has reported that PS^+^ EVs can directly bind to and activate CD8^+^ T cells, driving their differentiation and proliferation during antiviral immune surveillance in mice (114). Thus, dysregulation of PS^+^ EVs may contribute to autoimmune pathogenesis both by impairing macrophage-dependent clearance of immunogenic dead cell debris and by promoting autoreactive T cell responses.

The CD47–signal-regulatory protein α (SIRPα) interaction serves as a critical phagocytosis checkpoint, where CD47-expressing cells deliver a “don’t-eat-me” signal through SIRPα on myeloid cells to maintain immune homeostasis (173). This pathway has significant clinical implications, as CD47 enables both tumor (174) and apoptotic (139) cells to evade immune surveillance. Therapeutic blockade of CD47 enhances phagocytic clearance and demonstrates protective effects in murine models of autoimmune encephalomyelitis (175) and lupus nephritis (176). Pathologically, CD47-carrying exosomes can contribute to disease progression by suppressing macrophage-mediated clearance of apoptotic or cancer cells (177), thereby exacerbating autoimmune pathogenesis (175, 176) and promoting tumor immune evasion (177). Leveraging these immune-evasive properties, CD47-bearing EVs have been used for the targeted delivery of cyclosporin A/miR inhibitors, protecting mice from hepatic ischemia/reperfusion injury (135). This highlights their potential as a sophisticated tool for precisely modulating immune surveillance in a clinical setting.

The PD-1/PD-L1 axis is a critical immune checkpoint that delivers “don’t-kill-me” inhibitory signals to regulate T cell responses and maintain immune tolerance. The clinical importance of this pathway is highlighted by the development of lupus-like autoimmunity in PD-1–deficient mice, which exhibit uncontrolled T cell activity (178). In oncology, PD-L1–bearing exosomes or EVs from patients with melanoma (179) or glioblastoma (180) have been shown to mediate immune evasion by suppressing antitumor T cells. Similarly, in mice PD-L1–bearing EVs promote tumor progression (162). In an autoimmune context, higher levels of PD-L1–bearing β cell–derived EVs correlate with a greater number of remaining β cells in patients with type 1 diabetes, suggesting a protective role in preserving β cell mass, as these EVs have been shown to inhibit CD8^+^ T cell activity in vitro (181). PD-L1–expressing MSC-EVs exhibit suppressive effects on T cell activation and ameliorate psoriatic inflammation in mouse models (182).

Furthermore, CD73-carrying EVs from Tregs have been shown to suppress T cell proliferation by generating immunosuppressive adenosine (63, 93). Exosomes from tumor cells and those circulating in the blood of patients with cancer carry NKG2D ligands that have been shown to suppress NK and CD8^+^ T cell cytotoxicity by downregulating NKG2D, enabling cancer cells to evade immune surveillance (183, 184). The clinical significance of this pathway is highlighted by the detection of NKG2D ligand^+^ MVs in the bone marrow of patients with multiple myeloma (185), suggesting their pivotal role in creating an immunosuppressive microenvironment that favors tumor growth. Additionally, EVs can also modulate immune surveillance by interacting with the complement system (186), which is often associated with the EV protein corona (50, 51).

In summary, emerging evidence indicates that EVs may regulate immune surveillance through multiple mechanisms, such as (a) activating cytosolic surveillance sensors (150, 151, 187), (b) modulating immune checkpoints (179, 180, 188), (c) regulating NK/T cell cytotoxicity (152, 156), and (d) influencing macrophage and DC phagocytic clearance (171, 172, 175, 176). Notably, the field remains critically underexplored. Further research into immune surveillance in autoimmune diseases and the involvement of EVs is required to pave the way for innovative therapeutic strategies against immune dysregulation.

## Conclusions and challenges

As potential mediators linking cellular processes and autoimmune pathogenesis, EVs are recognized to transport cargo from distinct subcellular compartments, contributing to disease progression by delivering immunogenic molecules, regulating antigen processing and presentation, and modulating various immune responses. EVs can fuel inflammation by carrying proinflammatory mediators, sustaining autoimmune responses and promoting end-organ damage. Emerging evidence also suggests that EVs might regulate immune surveillance by influencing cytosolic sensors, immune checkpoints, NK/T cell cytotoxicity, and macrophage/DC phagocytic activity. However, the precise role of EV-mediated immune surveillance in maintaining immune homeostasis and driving autoimmunity remains a critical frontier for further research, requiring rigorous basic and clinical investigation to delineate the characteristics of autoimmunity from those observed over the past decades in cancer biology.

Despite significant progress over the past decades, EV research continues to face considerable technical hurdles in functional studies and establishing causality. The general advantages, disadvantages, and technical challenges in EV research have been thoroughly discussed elsewhere (189). In this Review, we focused on emerging mechanistic and translational findings to highlight critical knowledge gaps in the context of autoimmune diseases. Although existing studies have employed a variety of EV isolation and detection techniques (Tables 1–3), key issues remain in EV functional studies, including (a) contamination by nonvesicular particles, (b) inherent complexity due to EV population heterogeneity, and (c) a general lack of mechanistic proof with correlation studies (Table 5). Future studies must adopt standardized methodologies (i.e., MISEV2023) (189) and incorporate rigorous in vivo validation — specifically tackling key challenges, such as distinguishing EV effects from parental cell activities, controlling for intervention off-target effects, and improving our limited understanding of EV pharmacokinetics and biodistribution in different organs (Table 5). To establish robust causal conclusions, strategies like selectively blocking EV production, interrupting EV uptake in vivo, or performing cargo-specific modifications could be employed. Furthermore, high-throughput techniques like proteomics, RNA sequencing, and time-of-flight mass spectrometry will be helpful for translating findings from the bench to the bedside.

Key knowledge gaps in our understanding of EV biology still remain, including, but not limited to, the regulation of EV biogenesis, selective cargo sorting, and the content-dependent effects of EVs on various immune responses. This is further complicated by the dual pro- and antiinflammatory roles of EVs, which means their effects are highly dependent on context. These complexities necessitate the development of tailored therapeutic strategies for future clinical applications. Beyond targeting natural EVs, engineered EVs have emerged as promising platforms for targeted therapy. As discussed elsewhere (190, 191), significant translational hurdles remain for EV-based therapeutics, including scalable production, storage and in vivo stability, appropriate dosing, and managing their immunogenicity and potential to disrupt immune balance. All of these challenges need to be addressed for successful clinical use. Despite these obstacles, EV research holds great promise for advancing our mechanistic understanding of autoimmune pathogenesis and fostering the development of innovative therapeutic strategies.

## Author contributions

MLL, WW, and XW conceived the concepts and designed the study. XW, YL, YZ, WW, and MLL drafted the first version of the manuscript. YZ, XL, XW, YL, NZ, WW, and MLL critically revised and finalized the manuscript. All authors critically contributed important intellectual content to the manuscript.

## Funding support

- NIH (grant R21AI144838).
- Lupus Research Alliance (grant 416805).

## Figures and Tables

**Table 4:**
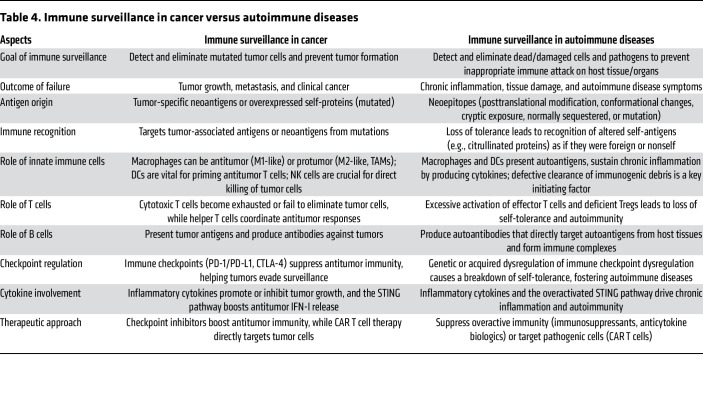
Immune surveillance in cancer versus autoimmune diseases

**Table 3:**
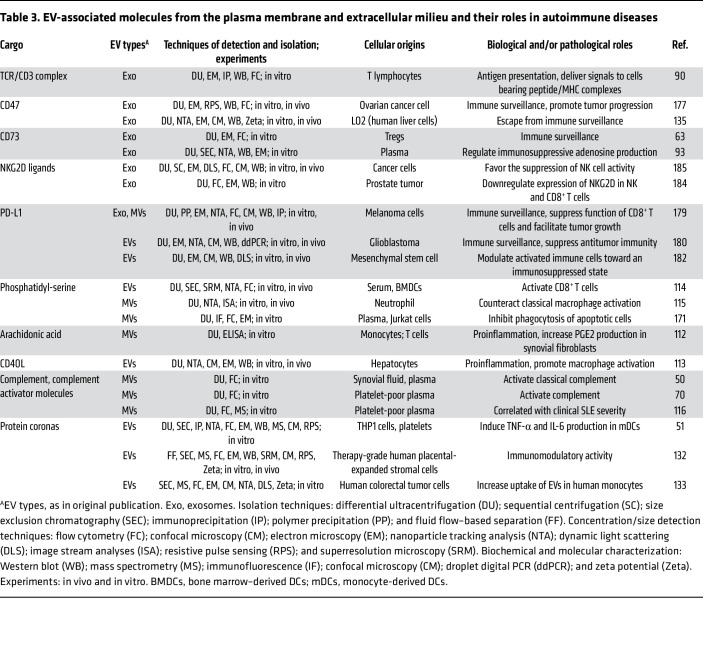
EV-associated molecules from the plasma membrane and extracellular milieu and their roles in autoimmune diseases

**Table 2:**
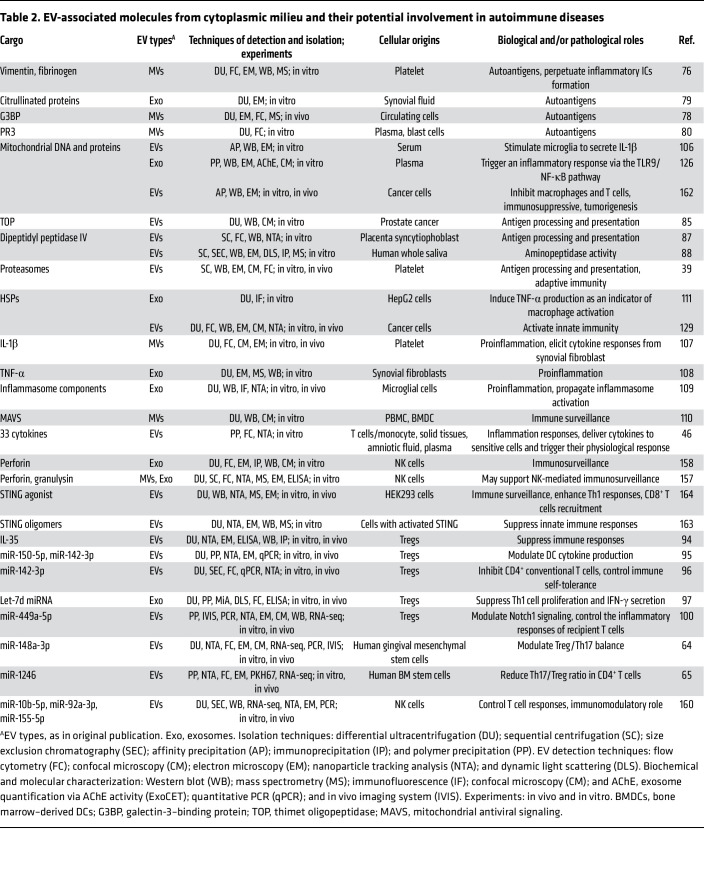
EV-associated molecules from cytoplasmic milieu and their potential involvement in autoimmune diseases

**Table 1:**
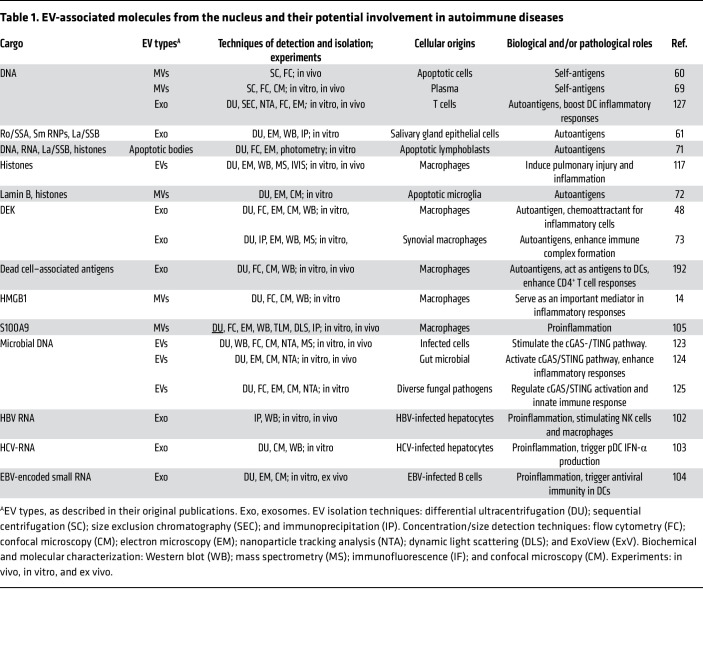
EV-associated molecules from the nucleus and their potential involvement in autoimmune diseases

**Table 5:**
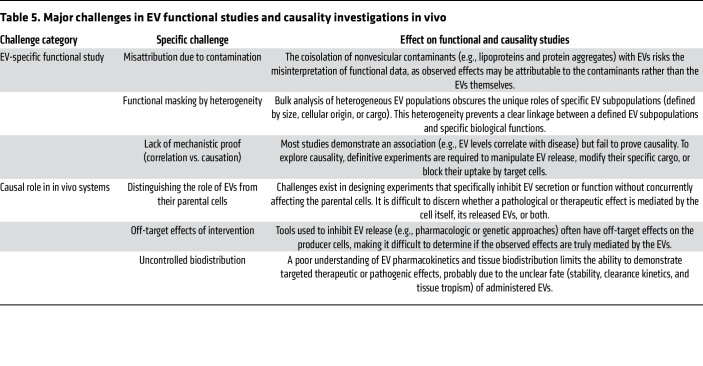
Major challenges in EV functional studies and causality investigations in vivo

## Version 1 02/02/2026

Electronic publication
